# 

**Published:** 1888-05

**Authors:** 


### CONTENTS, MAY, 1888 (VOL. III, NO. XI.)

ORIGINAL ARTICLES.—The Circulation in the Kidney--By Bat. Smith, M. D., Wharton ........ 459

A Case of Typhlitis Stercoralis—By S. L. Post, M. D., Gainesville ........ 462

The Influence of Heredity in the Production of Crime; and the Punishment of Hereditary or Confirmed Criminals—By W. M. Yandall, M. D., El Paso ........ 464

CORRESPONDENCE.—A Newsy Letter from San Antonio; the New Medical College, etc.; *Medicus* ........ 470

SOCIETY NOTES.—A Synopsis of Proceedings of the Texas State Medical Association ........ 473

Austin District Medical Society ........ 484

The Physicians' Mutual Benefit Association of Texas ........ 485

The North Texas Medical Association ........ 497

The Lone Star Medical Association ........ 486

The American Medical Association ........ 487

Cherokee County Medical Society ........ 487

The Texas Pharmaceutical Association ........ 487

EDITORIALS.—The Late Meeting Texas State Medical Association ........ 489

The Effort to Secure a Room in the Capitol for the Use of the Texas State Mddical Association ........ 491

Dr. J. F. X. Paine, President-elect of the Texas State Medical Association ........ 492

BOOK NOTICES.—The University Magazine ........ 496

Lesions of the Pelvic Floor—By B. E. Hadra, M. D., Austin 495

MEDICAL NEWS AND MISCELLANY.—Personals—Removals—The Texas Medical College, Faculty, etc ........ 497–8

A Few Remarks to the Point ........ 498

Straining at a Gnat and Swallowing a Camel ........ 499

